# Domestic Summary

**Published:** 1838-09-26

**Authors:** 


### DOMESTIC SUMMARY.

*Medical School of Yale College.*—Professor Knight has been transferred to the Chair of Surgery, in this institution, vacated by the death of Professor Hubbard; and Dr. Charles Hooker, of New Haven, has been appointed to the Chair of Anatomy and Physiology.

*Hydrated Peroxide of Iron, as an Antidote for Arsenic.*—Dr. Robert B. Hall, of Virginia, has reported, in the twelfth number of the American Medical Intelligencer, some experiments with the arsenious acid and the peroxide of iron, upon dogs. His conclusions are:—the arsenious acid is innocuous upon the economy of the dog, when given in doses sufficiently large to produce vomiting; if the poison be retained, either by a ligature upon the œsophagus, or by doses too minute to excite emesis, it is certainly fatal. The hydrated iron *is* an antidote.

We have received a Discourse on the importance of a general diffusion of a knowledge of Anatomy, Physiology, and Hygiene, delivered at the Auburn Female Seminary, in May last, by Dr. F. H. Hamilton. The discourse was introductory to a course of lectures on the subjects mentioned. Attempts like these, to popularize medicine, are highly commendable; and we believe that much might be accomplished in resisting the advances of quackery, if the physicians of the country generally would devote themselves to similar efforts. Dr. Hamilton's address is a production of much merit.

*Dislocations of the Shoulder.*—In the London Lancet, for August 4th, is reported a reduction of a dislocation of the humerus under the pectoral muscle, of twenty-eight days' standing, "as a parallel to the case transcribed into the Lancet of June 16th, from the Medical Examiner, of the reduction of a luxation of thirty-one days' standing." A report of the reduction of a luxation of the humerus, of fifty-one days' standing, by Dr. T. Harris, is contained in one of our early numbers.

